# Dexamethasone accelerates muscle regeneration by modulating kinesin-1-mediated focal adhesion signals

**DOI:** 10.1038/s41420-021-00412-4

**Published:** 2021-02-17

**Authors:** Jong-Wei Lin, Yi-Man Huang, Yin-Quan Chen, Ting-Yun Chuang, Tien-Yun Lan, Yen-Wenn Liu, Hung-Wei Pan, Li-Ru You, Yang-Kao Wang, Keng-hui Lin, Arthur Chiou, Jean-Cheng Kuo

**Affiliations:** 1 Institute of Biochemistry and Molecular Biology, National Yang Ming Chiao Tung University, Hsinchu, 30010 Taiwan; 2 Cancer Progression Research Center, National Yang Ming Chiao Tung University, Hsinchu, 30010 Taiwan; 3 Institute of Biophotonics, National Yang Ming Chiao Tung University, Hsinchu, 30010 Taiwan; 4 School of Medicine for International Students, College of Medicine, I-Shou University, Kaohsiung, 82445 Taiwan; 5 Department of Cell Biology and Anatomy, College of Medicine, National Cheng Kung University, Tainan, 70101 Taiwan; 6 Institute of Physics, Academia Sinica, Taipei, 11529 Taiwan

**Keywords:** Kinesin, Focal adhesion

## Abstract

During differentiation, skeletal muscle develops mature multinucleated muscle fibers, which could contract to exert force on a substrate. Muscle dysfunction occurs progressively in patients with muscular dystrophy, leading to a loss of the ability to walk and eventually to death. The synthetic glucocorticoid dexamethasone (Dex) has been used therapeutically to treat muscular dystrophy by an inhibition of inflammation, followed by slowing muscle degeneration and stabilizing muscle strength. Here, in mice with muscle injury, we found that Dex significantly promotes muscle regeneration via promoting kinesin-1 motor activity. Nevertheless, how Dex promotes myogenesis through kinesin-1 motors remains unclear. We found that Dex directly increases kinesin-1 motor activity, which is required for the expression of a myogenic marker (muscle myosin heavy chain 1/2), and also for the process of myoblast fusion and the formation of polarized myotubes. Upon differentiation, kinesin-1 mediates the recruitment of integrin β1 onto microtubules allowing delivery of the protein into focal adhesions. Integrin β1-mediated focal adhesion signaling then guides myoblast fusion towards a polarized morphology. By imposing geometric constrains via micropatterns, we have proved that cell adhesion is able to rescue the defects caused by kinesin-1 inhibition during the process of myogenesis. These discoveries reveal a mechanism by which Dex is able to promote myogenesis, and lead us towards approaches that are more efficient in improving skeletal muscle regeneration.

## Introduction

Skeletal muscle fibers (muscle cells) develop when the myogenic program is activated resulting in a repression of cell proliferation and a stimulation of differentiation^1–3^. During muscle differentiation, several steps which change the structural and physiological functioning of the cytoskeleton are involved. These result in the evolution of single cells into multinucleated muscle fibers. In detail, myoblasts form cell adhesion organelles, namely focal adhesions (FAs), by engagement with the extracellular matrix (ECM); the later (FAs) enable the myoblasts to align with each other. After this, their actin filaments, which are anchored to the FAs, begin to form the myofibrils. During the next step, the differentiated myocytes fuse with each other to form multinucleated myotubes that contain strong myofibrils, with contractile capacity, essential for the muscle to function^4–10^. The main purpose of muscle fibers is to develop force by contracting relative to their surrounding ECM, such that the human body and other animals could support themselves and move.

The contractile apparatus in muscle fibers is driven by cytoskeleton structures^11,12^. Actin filaments interact with FAs to generate force and they serve as the major functional cytoskeletal component within a muscle fiber^13–15^. FAs start to form when integrin receptors engage with the ECM and begin to be activated. Next, a series of FA-associated proteins are recruited to connect with the actin cytoskeleton^16–21^. Under biochemical stimuli, as well as physical stimuli, the size and composition of FAs are regulated spatio-temporally in a process called FA maturation^22–26^. Modulation of the maturation state of the FAs plays a key determinant role in the biological responses downstream of integrin engagement, such as cell mobility^27^, stem cell differentiation lineage determination^28^, and myogenic differentiation^29^. The fact that integrin β1 is required for myoblast fusion has been demonstrated both in vivo and in vitro^30^. In addition, these integrin-mediated signals, via focal adhesion kinase (FAK) activation, have been shown to play crucial roles in myoblast fusion in vitro and in muscle regeneration in vivo^29^. Therefore, FA organization is directly involved in the transmission of integrin-mediated signaling, which, in turn, plays a crucial role in controlling the process of muscle differentiation.

The microtubules present in muscle cells are extensively remodeled, and aligned parallel to the major cell axis; this orientation is central to determine the overall shape of a cell during myogenesis. Microtubule motor protein KIF5B, the expressed isoform of kinesin-1 heavy chain, which is abundant in skeletal muscle^31^, has been demonstrated to be responsible for muscle development^32^. A study using mice with *Kif5b* conditionally knocked out in skeletal muscles shows that loss of kinesin-1 causes the aggregation of nuclei, mitochondria, and myofibril components, including α-sarcomeric actin and non-muscle myosin IIB, within the cell body. This, in turn, results in impaired myofibrils assembly and muscle fiber terminal disturbances, leading to lateral detachment of myofibrils from the sarcolemma, with integrins still present on the sarcolemma^32^. This reveals the connection between integrins and actin filaments lost due to the loss of kinesin-1. It implies that the purpose of kinesin-1 is to maintain the structural organization of the integrin-mediated adhesion organelles FAs in skeletal muscles to retain the integration of myofibrils to integrins at sarcolemma. Other studies have indicated that, in normal skeletal muscle, integrin β1 and various FA-associated proteins, including paxillin, talin, and vinculin, localize at the myotendinous junctions and form physical links between the sarcomeric units and the sarcolemma^33^. These findings support the idea that kinesin-1 controls FA formation and the transmission of integrin-mediated FA signals during myogenesis. However, whether kinesin-1 regulates myogenesis through integrin-mediated FA signaling has never been investigated.

Muscle function loss happens in people with muscular dystrophy, the main clinical features of which are progressive loss of skeletal muscle and prominent muscle inflammation^34–40^; these eventually lead to the loss of the ability to walk and then death. To date, there is no treatment available to stop or to reverse any type of muscular dystrophy. Nevertheless, the synthetic glucocorticoid, such as dexamethasone (Dex), has been applied therapeutically to slow down muscle degeneration and stabilize muscle strength^41,42^. In addition, Dex seems to enhance the myogenic fusion efficiency of mouse myoblasts C2C12 cells^43^ via unknown mechanisms. Here, in the present study, we have demonstrated that Dex is sufficient to promote muscle regeneration in vivo and that this is mediated via kinesin-1 motor activity. We sought to investigate whether Dex regulates kinesin-1 motor activity, and further explored whether integrin β1-mediated FA signaling is related to kinesin-1 in promoting myogenic differentiation.

## Results

### Dexamethasone enhances kinesin-1 motor activity

Previous studies have shown that Dex enhances myogenic differentiation^43^. To confirm this effect, C2C12 cells were exposed to differentiation media containing the indicated concentrations of Dex for 0–5 days, and the expression of the myogenic marker myosin heavy chain 1/2 (MYH1/2) was accessed. Dex treatment induced expression of MYH1/2 during the early phase of the C2C12 differentiation time course (Supplemental Fig. 1a), indicating that Dex facilitated myogenic differentiation. On Day 5 of the time course, cells were stained to detect MYH1/2 and with DAPI to examine whether Dex promoted the fusion of mono-nuclear cells to form multi-nuclei myotubes (Supplemental Fig. 1b). By counting the number of nuclei per MYH1/2+ cell, we quantified myogenic fusion efficiency (fusion index). Higher myogenic fusion percentage was identified in the cells treated with Dex (Supplemental Fig. 1c). This confirmed that Dexinduced myogenic differentiation of C2C12 cells.

**Fig. 1:**
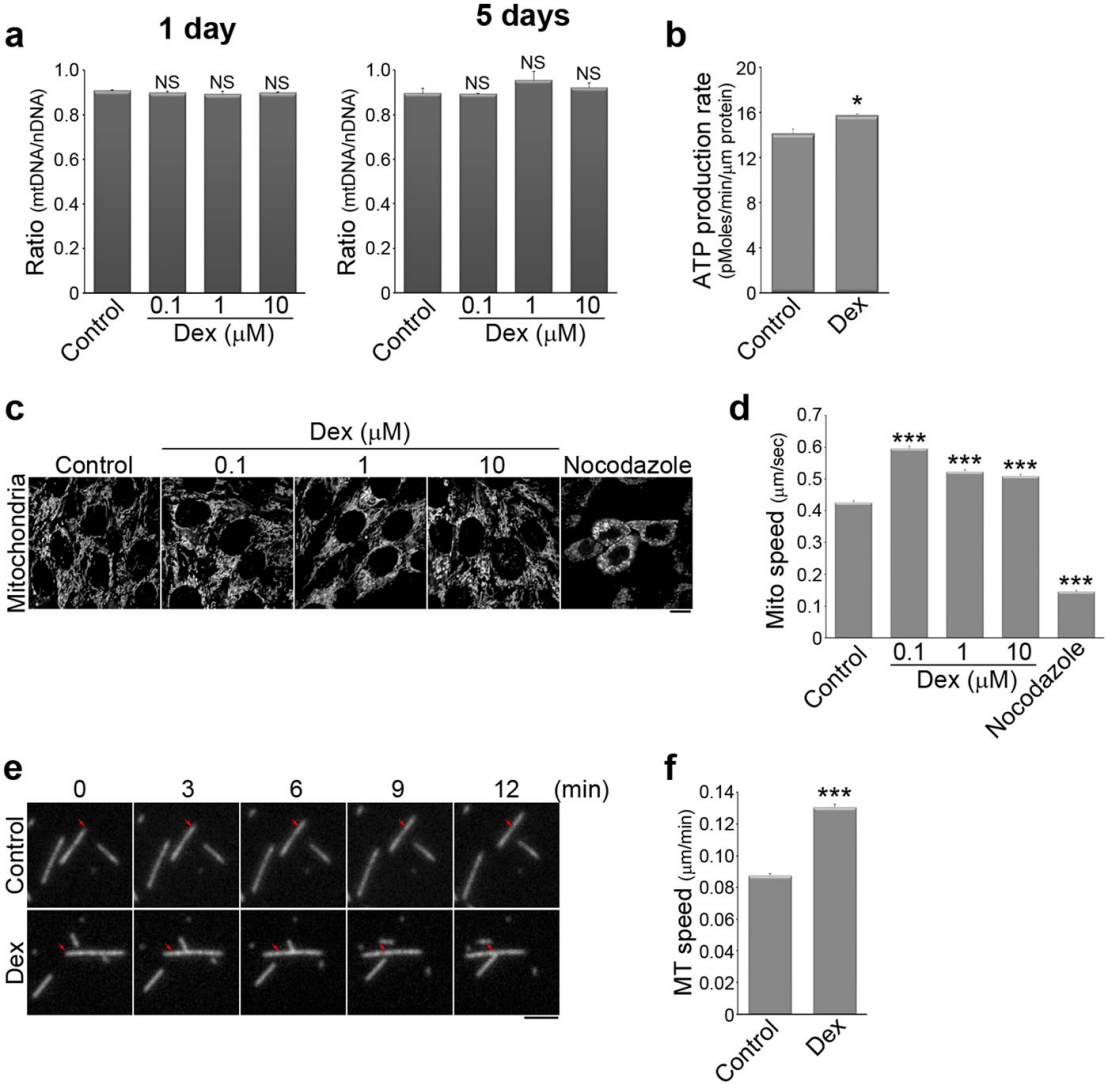
Dexamethasone promotes mitochondria dynamics and enhances kinesin-1 motor activity. **a** C2C12 cells treated with DMSO (control), 0.1, 1, or 10 µM Dex in differentiation medium for 1 day or 5 days had their mitochondrial numbers assessed using the ratio of cytochrome C oxidase I DNA (to indicate mitochondrial DNA; mtDNA) to 18S rRNA DNA (to indicate nuclear DNA; nDNA) by real-time PCR. Data are mean ± s.e.m (*n* = 3 independent experiments). NS no significance (compared with the control). **b** ATP production rate was calculated from oxygen consumption rate (OCR) in C2C12 cells treated with DMSO (control) or 1 µM Dex for 6 h. OCR was measured continuously followed by the addition of oligomycin (1 µM), Carbonyl cyanide 4-(trifluoromethoxy) phenylhydrazone (FCCP; 1 µM), and antimycin A (0.5 µM) with rotenone (0.5 µM) using Seahorse XF^e^24 Extracellular Flux Analyzer. Data are mean ± s.e.m (*n* = 3 independent experiments). *p < 0.05 (compared with control). **c** C2C12 cells treated with DMSO (control), 0.1 µM Dex, 1 µM Dex, 10 µM Dex for 1 h, or with 10 µM nocodazole for 16 h were stained with MitoTracker Red to visualize mitochondria. Scale bar, 10 µm. **d** Comparison of the average values for the speed of mitochondrial movement in C2C12 cells. Data are mean ± s.e.m (control, *n* = 3638 mitochondria/5 cells; 0.1 µM Dex, *n* = 5016 mitochondria/6 cells; 1 µM Dex, *n* = 3635 mitochondria/6 cells; 10 µM Dex, *n* = 4818 mitochondria/6 cells; nocodazole, *n* = 632 mitochondria/2 cells). ***p < 0.001 (compared with the control). **e** Time-lapse epi-fluorescence microscopy showing the movement of rhodamine-labeled microtubules treated with control (DMSO) or 1 µM Dex, using in vitro kinesin-1 motility assay. Scale bar, 2 µm. **f** Comparison of the average values for the speed of microtubule movement brought about by kinesin-1. Data are mean ± s.e.m (control, *n* = 494 microtubules; 1 µM Dex, *n* = 370 microtubules). ***p < 0.001 (compared with control).

Mitochondria activity is important for myogenic differentiation^44^, and therefore we sought to determine the effect of Dex on the mitochondria function in the C2C12 cells. We first examined whether Dex changed the abundance of mitochondria present during myogenic differentiation. To do this, we measured the ratio of cytochrome C oxidase I DNA (to indicate mitochondrial DNA; mtDNA) to 18S rRNA DNA (to indicate nuclear DNA; nDNA) in C2C12 cells that had been cultured in differentiation media with the indicated concentrations of Dex for 1 or 5 days. We found that Dex did not influence the abundance of mitochondria during myogenic differentiation (Fig. 1a). However, we detected higher ATP production in C2C12 cells treated with Dex by measuring oxygen consumption rate (OCR) using Seahorse XF24 Extracellular Flux analysis (Fig. 1b). Mitochondria are highly dynamic organelles in the cells^45^. Evidence has indicated that changing mitochondrial motility often affects signaling pathways and cell functioning in many different ways^46^. We next examined whether Dex affected mitochondrial dynamics in C2C12 cells. We performed live-cell imaging using MitoTracker Red to label the mitochondria of living cells (Fig. 1c). Using a time-lapse image series that showed the presence of MitoTracker Red in C2C12 cells, we tracked mitochondrial motion using a previously developed software package, namely Mytoe^47^. The results allowed us to quantify the movement speed of individual mitochondria. Compared to the control cells, Dex-treated cells showed significantly higher mitochondrial speeds (Fig. 1d). Thus, Dex stimulates the mitochondria in C2C12 cells and increases their motility.

Microtubule motor kinesin-1 (also call KIF5B or conventional kinesin) is known to drive mitochondrial movement along microtubules^48,49^. We confirmed that depolymerization of microtubules by nocodazole significantly reduced the movement speed of the mitochondria (Fig. 1c, d). Therefore, we hypothesized that Dex affected kinesin-1 motors, which led to faster mitochondrial movement and myogenic differentiation. We therefore used an in vitro kinesin-1 motility assay with fluorescence-labeled microtubules (rhodamine-labeled purified tubulin) added onto the surface of coverslips containing coupled kinesin-1 motors. Using time-lapse epi-fluorescent image series of the rhodamine-labeled microtubules, the kinesin-1-induced motility of the microtubules was tracked (Fig. 1e). The results revealed that Dex significantly increased the speed of kinesin-1 as it moved along microtubules. The increase was from ~0.088 µm/min (0.088 µm/min ± 0.001) to ~0.131 µm/min (0.131 µm/min ± 0.002; Fig. 1f). These results show that Dex increases kinesin-1 motor activity.

### Dexamethasone enhances muscle regeneration in vivo through kinesin-1

**Fig. 2:**
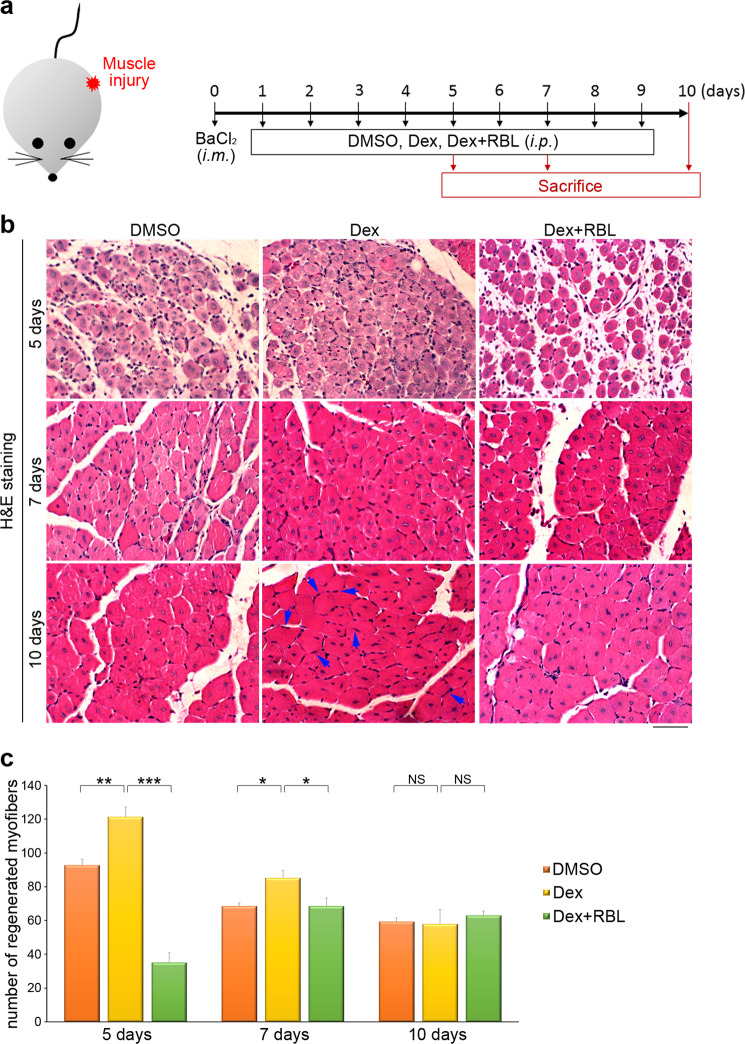
Kinesin-1 drives muscle regeneration in mice. **a** Experimental scheme for the skeletal muscle regeneration experiments. The tibialis anterior (TA) muscles in the left hind legs of mice were injured using 1.2% BaCl_2_ via intramuscular (i.m.) injection on day 0. Next, the mice were randomly grouped and administered with DMSO, Dex (0.1 mg/kg) or Dex (0.1 mg/kg) + RBL (30 mg/kg) everyday via intraperitoneal (i.p.) injection. To assess whether Dex is able to accelerate skeletal muscle regeneration via kinesin-1, mouse TA muscle samples were collected at various time points. **b** Representative histology images of mouse TA muscles of the various study groups. The regenerating myofibers with peripheral nuclei marked with blue arrows. Scale bar, 50 μm. **c** The number of regenerative muscle fibers, calculated as the number of fibers with accumulation of centrally located nuclei, as shown in **b**. Data are mean ± s.e.m (*n* = 5 independent fields). *p < 0.05; **p < 0.01; ***p < 0.001; NS no significance.

We next sought to determine whether Dex activates kinesin-1 motors, and further affects skeletal muscle regeneration in vivo. We established a BaCl_2_-induced muscle injury model using 9-week-old C57BL/6 female mice. The mice with their tibialis anterior (TA) muscles in the left hind legs injured (by 1.2% BaCl_2_) were treated with DMSO (the carrier control), Dex or Dex+ Rose Bengal Lactone (kinesin-1 inhibitor; RBL) everyday (Fig. 2a). The centrally nucleated myofibers, indicative of muscle regenerating were counted in H&E staining of TA muscle transverse sections (Fig. 2b). Compared with DMSO-treated mice, the Dex-treated mice showed a significantly larger number of centrally nucleated myofibers at 5 and 7 days post injury. At 10 days after injury, we observed that, in mice treated with Dex, most regenerating myofibers contained peripheral nuclei, indicating the role of Dex in promoting the formation and maturation of myofibers. In order to confirm that kinesin-1’s motor activity mediates the effect of Dex during muscle regeneration, we used an kinesin-1 inhibitor, RBL, to disrupt the association between kinesin-1 and microtubules^50,51^. The combined treatment of Dex and RBL significantly reduced the number of centrally nucleated myofibers at 5 and 7 days post injury, compared with Dex-treated mice, which supports the hypothesis that disrupting kinesin-1 motor activity retards the improved skeletal muscle regeneration caused by Dex treatment (Fig. 2c). These findings confirm that Dex activates kinesin-1 in skeletal muscle to promote myofiber regeneration after muscle injury, and highlight the importance of exploring Dex-activated kinesin-1 in promoting myogenic differentiation.

### The acceleration of myotube differentiation by dexamethasone requires kinesin-1 sliding along microtubules

**Fig. 3:**
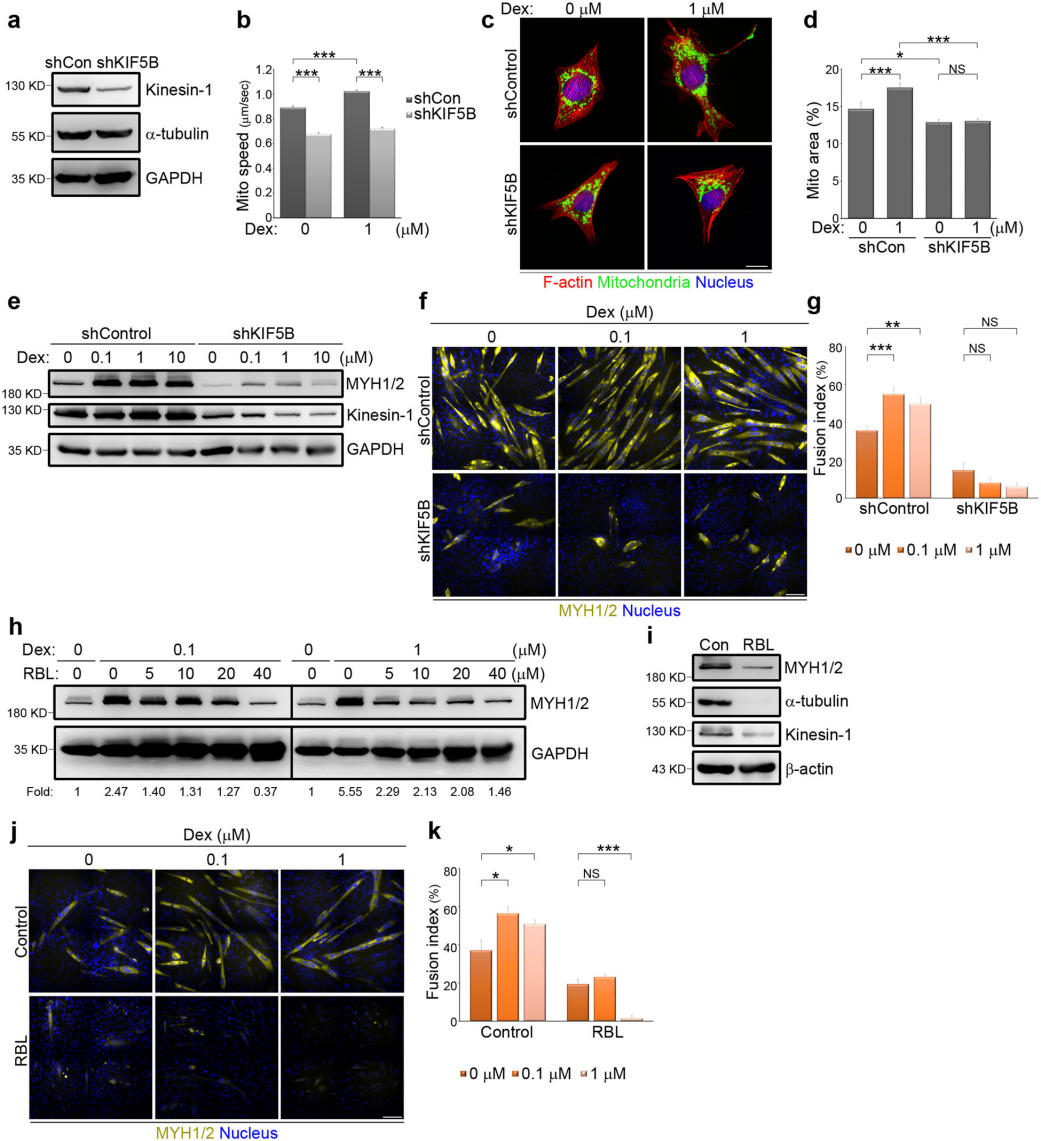
Kinesin-1 controls dexamethasone-promoted myogenic differentiation. **a** Total cell lysate from C2C12 cells expressing non-silenced (shCon) or Kinesin-1-silenced (shKIF5B) shRNAs were analyzed by western blotting using antibodies against kinesin-1, α-tubulin, and GAPDH. **b** Effects of kinesin-1-silencing on Dex-enhanced mitochondria dynamics. C2C12 cells expressing non-silenced (shCon) or Kinesin-1-silenced (shKIF5B) shRNAs were treated with the indicated concentrations of Dex for 1 h. Data are mean ± s.e.m (shCon in 0 µM Dex, *n* = 3481 mitochondria/6 cells; shCon in 1 µM Dex, *n* = 4716 mitochondria/6 cells; shKIF5B in 0 µM Dex, *n* = 3847 mitochondria/6 cells; shKIF5B in 1 µM Dex, *n* = 3578 mitochondria/6 cells). ***p < 0.001. **c** C2C12 cells expressing non-silenced (shControl) or Kinesin-1-silenced (shKIF5B) shRNAs were treated with the indicated concentrations of Dex for 6 h and immunostained for Tom20 (green; to visualize mitochondria), phalloidin (red; to visualize F-actin), and DAPI (blue; to visualize nucleus). Scale bar, 10 µm. **d** The mitochondria area (marked with Tom20) as a percentage within a cell (marked with phalloidin), as shown in **c**. Data are mean ± s.e.m (shCon in 0 μM Dex, *n* = 29 cells; shCon in 1 μM Dex, *n* = 30 cells; shKIF5B in 0 μM Dex, *n* = 30 cells; shKIF5B in 1 μM Dex, *n* = 30 cells). *p < 0.05; ***p < 0.001; NS no significance. **e** Effects of kinesin-1 silencing on Dex-enhanced myogenic differentiation. C2C12 cells expressing non-silenced (shControl) or Kinesin-1-silenced (shKIF5B) shRNAs were treated with differentiation medium containing the indicated concentrations of Dex for 3 days and analyzed by western blotting using antibodies against MYH1/2, kinesin-1, and GAPDH. **f** Effects of kinesin-1 on Dex-promoted myoblast fusion. C2C12 cells expressing non-silenced (shControl) or Kinesin-1-silenced (shKIF5B) shRNAs were treated with differentiation medium containing the indicated concentrations of Dex for 5 days and immunostained for MYH1/2 (yellow; myotube marker) and DAPI (to visualize nucleus). Scale bar, 100 µm. **g** Fusion index, calculated as the percentage of nuclei (≥3) in MYH1/2^+^ cells, as shown in **f**. Data are mean ± s.e.m (shControl in 0 µM Dex, *n* = 313 MYH1/2^+^ cells; shControl in 0.1 µM Dex, *n* = 236 MYH1/2^+^ cells; shControl in 1 µM Dex, *n* = 192 MYH1/2^+^ cells; shKIF5B in 0 µM Dex, *n* = 104 MYH1/2^+^ cells; shKIF5B in 0.1 µM Dex, *n* = 84 MYH1/2^+^ cells; and shKIF5B in 1 µM Dex, *n* = 79 MYH1/2^+^ cells). **p < 0.01; ***p < 0.001; NS no significance. **h** Effects of the association between kinesin-1 and microtubules on Dex-enhanced myogenic differentiation. C2C12 cells were treated with differentiation medium containing the indicated concentrations of Dex and kinesin-1 inhibitor RBL for 3 days and analyzed by western blotting using antibodies against MYH1/2, and GAPDH (internal control). The ratio of MYH1/2 to GAPDH is shown as fold. **i** C2C12 cells were treated with differentiation medium containing 40 µM RBL for 3 days and analyzed by western blotting using antibodies against MYH1/2, α-tubulin, kinesin-1, and β-actin. **j** Effects of the association between kinesin-1 and microtubules on Dex-promoted myoblast fusion. C2C12 cells were treated with differentiation medium containing the indicated concentrations of Dex accompanied with either DMSO (control) or 40 µM RBL for 5 days and immunostained for MYH1/2 (yellow; myotube marker) and DAPI (to visualize nucleus). Scale bar, 100 µm. **k** Fusion index, calculated as the percentage of nuclei (≥3) in MYH1/2^+^ cells, as shown in **j**. Data are mean ± s.e.m (DMSO in 0 µM Dex, *n* = 144 MYH1/2^+^ cells; DMSO in 0.1 µM Dex, *n* = 160 MYH1/2^+^ cells; DMSO in 1 µM Dex, *n* = 157 MYH1/2^+^ cells; RBL in 0 µM Dex, *n* = 130 MYH1/2^+^ cells; RBL in 0.1 µM Dex, *n* = 164 MYH1/2^+^ cells; RBL in 1 µM Dex, *n* = 34 MYH1/2^+^ cells). *p < 0.05; ***p < 0.001; NS no significance.

To examine the direct effect of Dex on kinesin-1 motors in promoting myogenic differentiation, we generated non-silenced and kinesin-1-silenced C2C12 cells using a lentivirial short hairpin RNA (shRNA) (Fig. 3a). We first examined whether the observed rapid mitochondrial movement induced by Dex (Fig. 1c, d) was due to a direct up-regulation of kinesin-1 motor activity. These two types of cells were stained with MitoTracker Red in order to monitor the movement of individual mitochondria. We found that the increase in mitochondrial speed brought about by treatment with Dex was absent in the kinesin-1-silenced cells (Fig. 3b). When assessed using the mitochondrial stain Tom 20, we observed that treatment with Dex led to a dramatic redistribution of the mitochondria towards the cell periphery in non-silenced cells. Furthermore, depletion of kinesin-1 was able to suppress this effect of Dex, resulting in a marked accumulation of mitochondria close to the nucleus (Fig. 3c). By quantifying the area of mitochondria spreading as a percentage of each cell, we confirmed that Dex increased the area across which mitochondria were distributed in non-silenced cells, but not in the kinesin-1-silenced cells (Fig. 3d). Specifically, the depletion of kinesin-1 substantially shrank the area of spread of the mitochondria, as shown in Fig. 3d. These results confirm that Dex affects mitochondrial movement by directly promoting kinesin-1 motors activity.

Given that inhibition of microtubule depolymerization with nocodazole (Supplemental Fig. 2) and depletion of kinesin-1 (Fig. 3e) inhibited myogenic differentiation, we sought to determine the role of kinesin-1 in Dex-mediated myogenic differentiation. Non-silenced and kinesin-1-silenced cells were exposed to differentiation media containing the indicated concentrations of Dex. Then, an analysis of MYH1/2 expression was carried out (Fig. 3e) in parallel with measurement of the myogenic fusion indices of the various treatment groups (Fig. 3f, g). We found that silencing of kinesin-1 significantly suppressed both MYH1/2 expression (Fig. 3e) and myocyte fusion in C2C12 cells that had been treated with Dex (Fig. 3f, g), which reveals the essential role that kinesin-1 plays in Dex-mediated myogenic differentiation. Subsequently, C2C12 cells were exposed for 3 days to differentiation media containing the indicated concentrations of Dex and kinesin-1 inhibitor RBL^50,51^. We found that RBL dramatically inhibited Dex-induced MYH1/2 expression in a dose-dependent manner (Fig. 3h). After being placed in the differentiation medium with 40 μM RBL for 3 days, protein down-regulation of kinesin-1 and α-tubulins was detected (Fig. 3i). We also measured the cells’ myogenic fusion indices, and confirmed that disruption of kinesin-1 moving along the microtubules caused a dramatic reduction in Dex-induced multi-nuclei myotubes formation (Fig. 3j, k). These results support the hypothesis that Dex affects myogenic differentiation via direct activation of kinesin-1 motors.

### Kinesin-1 is responsible for integrin β1 transportation, which acts as a focal adhesion signal

**Fig. 4:**
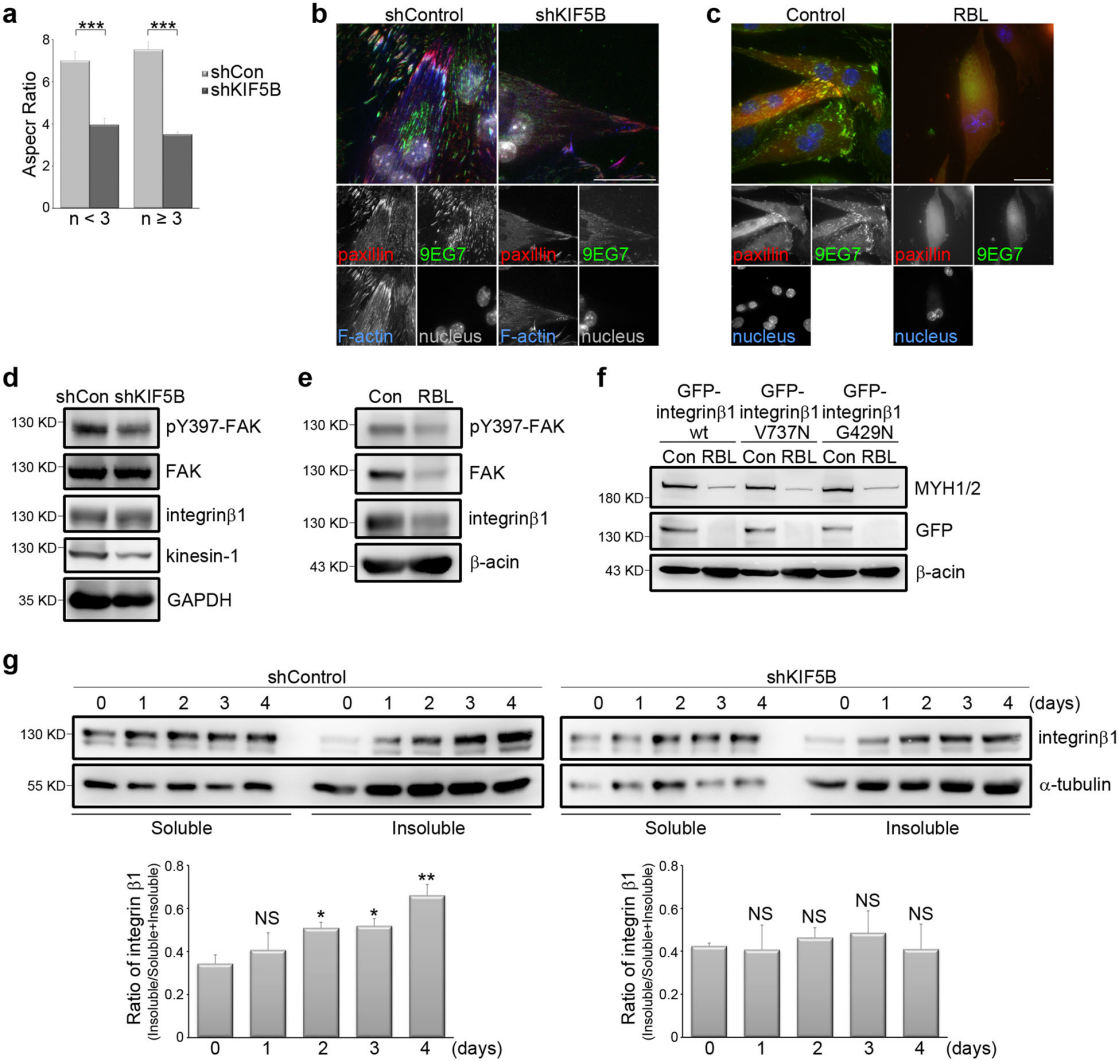
Kinesin-1 regulates integrin β1 activation and myotube morphology. **a** Effects of kinesin-1 expression on morphological changes of myotubes. C2C12 cells expressing non-silenced (shCon) or Kinesin-1-silenced (shKIF5B) shRNAs were treated with differentiation medium for 5 days and immunostained for MYH1/2 and DAPI. The MYH1/2^+^ cells were grouped based on the number (*n*) of nucleus in order to calculate the aspect ratio, namely the ratio of cell’s longest length to the cell’s shortest length. Data are mean ± s.e.m (shCon with <3 nuclei, *n* = 68 MYH1/2^+^ cells; shCon with ≥3 nuclei, *n* = 45 MYH1/2^+^ cells; shKIF5B with <3 nuclei, *n* = 168 MYH1/2^+^ cells; shKIF5B with ≥3 nuclei, *n* = 23 MYH1/2^+^ cells). ***p < 0.001. **b** TIRF microscopy images of immunolocalized paxillin (to visualize FAs; red), 9EG7 (to visualize activated integrin β1; green), phalloidin (to visualize F-actin; blue), and DAPI (to visualize nucleus; gray) in non-silenced (shControl) and kinesin-1-silenced (shKIF5B) C2C12 cells treated with differentiation medium for 5 days. Scale bar, 30 μm. **c** TIRF microscopy images of immunolocalized paxillin (to visualize FAs; red), 9EG7 (to visualize activated integrin β1; green), and DAPI (to visualize nucleus; blue) in C2C12 cells, treated with differentiation medium containing DMSO (Control) or 40 µM RBL for 5 days. Scale bar, 30 μm. **d** Western blot analysis of cell lysates obtained from non-silenced (shCon) and kinesin-1-silenced (shKIF5B) C2C12 cells, treated with differentiation medium for 3 days, using pY397-FAK, FAK, kinesin-1, integrin β1, and GAPDH antibodies. **e** Western blot analysis of cell lysates obtained from C2C12 cells treated with differentiation medium containing DMSO (Con) or 40 µM RBL for 3 days, using pY397-FAK, FAK, integrin β1, and β-actin antibodies. **f** Western blot analysis of cell lysates obtained from C2C12 cells expressing GFP-integrin β1 wt, GFP-integrin β1 V737N, or GFP-integrin β1 G429N, treated with differentiation medium containing DMSO (Con) or 40 µM RBL for 3 days, using MYH1/2, GFP, and β-actin antibodies. **g** Top: pellets (polymerized microtubule-containing fractions; insoluble) and supernatants (free tubulin fractions; soluble) from non-silenced (shControl) and kinesin-1-silenced (shKIF5B) C2C12 cells treated with differentiation medium for 0, 1, 2, 3, or 4 days were fractionated and then analyzed by western blotting using antibodies against integrin β1 and α-tubulin. Bottom: ratio of integrin β1 in the polymerized microtubule-containing fractions (pellet; insoluble): total (insoluble + soluble fractions) as determined by western blotting (*n* = 4 independent experiments). Data are mean ± s.e.m. *p < 0.05; **p < 0.01; NS, no significance, all compared with 0 day.

Kinesin-1 has been implicated in generating and stabilizing muscle cell organization in mice^32^. In kinesin-1-silenced C2C12 cells cultured for 5 days in differentiation media, we detected fewer cells that expressed the myogenic marker MYH1/2 compared to non-silenced cells (Fig. 3f). Of these kinesin-1-silenced C2C12 cells showing MYH1/2^+^ expression, we quantified their aspect ratio, and found that their aspect ratio was lower than that of non-silenced C2C12 cells; this was true for cells both with less than three nuclei and those with more than three nuclei (Fig. 4a). This points to the fact that the elongation of cells, which is related to fusion, was blocked in these kinesin-1-silenced cells, which in turn supports a previous observation that kinesin-1-deficient mouse muscle fibers display shrunken ends and lateral detachment from the sarcolemma^32^. Based on the above, we next focused on FAs; this was because these integrin-based adhesive structures are known to act as a key regulator of integrin activation, and it is quite possible that FAs are regulated by kinesin-1. We acquired TIRF images of differentiated C2C12 cells to show the FA marker paxillin and active integrin β1 (9EG7). In non-silenced cells, we observed strong clustering of active integrin β1 and paxillin to form fibrillar FAs, particularly at the edge of the myotubes, and these FAs connected to bundled actin filaments. In contrast, in the kinesin-1-silenced cells, there was very limited clustering of active integrin β1 and paxillin, and as a result there were only a few FAs present; furthermore, only weak actin filaments were formed (Fig. 4b). These results reveal that kinesin-1 expression controls both the activation and clustering of integrin β1, and thereby allowing FA formation in the differentiated C2C12 cells. A similar effect was observed when kinesin-1 motor activity was abolished by RBL treatment (Fig. 4c). We next sought to determine whether kinesin-1 plays a crucial role in regulating integrin β1-mediated signaling within FAs by measuring the level of FAK phosphorylation at tyrosine 397^52,53^. We found that both silencing of kinesin-1 and disruption of its motor activity by RBL, independently, led to a significant decrease in phosphorylation levels of FAK at tyrosine 397 in the differentiated C2C12 cells (Fig. 4d, e). These results support the hypothesis that kinesin-1, via its motor activity, regulates the transmission of the integrin β1-mediated signals that bring about FA assembly and maturation, and that this in turn controls the elongation of cells during the process of myogenic differentiation.

Considering the critical role of integrin β1 clustering in promoting FAK phosphorylation at tyrosine 397^52,53^, we next investigated whether disruption of kinesin-1, and the resulting myogenic defects, could be restored by activating integrin β1. C2C12 cells were transfected with wild-type integrin β1 (integrin β1 wt), auto-clustering integrin β1 (integrinβ1 V737N), or constitutively active integrin β1 (integrinβ1 G429N). We observed that RBL treatment inhibited MYH1/2 expression in integrin β1 wt-expressing cells after 3 days in differentiation media. However, expression of either integrin β1 V737N or integrin β1 G429N in C2C12 cells failed to rescue the RBL-induced myogenic defect (Fig. 4f). Dissociating kinesin-1 and microtubules by the use of RBL has been shown to cause protein down-regulation of kinesin-1 and α-tubulin (Fig. 3i). We also found protein down-regulation of exogenous integrin β1 wt, exogenous integrin β1 V737N, and exogenous integrin β1 G429N (Fig. 4f). These results suggest that, during myogenic differentiation, integrin β1 transport along microtubules may happen in association with kinesin-1. Indeed, upon myogenic induction, an increased level of integrin β1 in the polymerized microtubule-containing fraction (pellet; insoluble fraction) was detected using a previously published microtubule isolation method^54^ (Fig. 4g). However, this effect was abolished in the kinesin-1-silenced cells (Fig. 4g). These findings confirm that integrin β1 is transported along the microtubules through kinesin-1 during the process of myogenic differentiation.

**Fig. 5:**
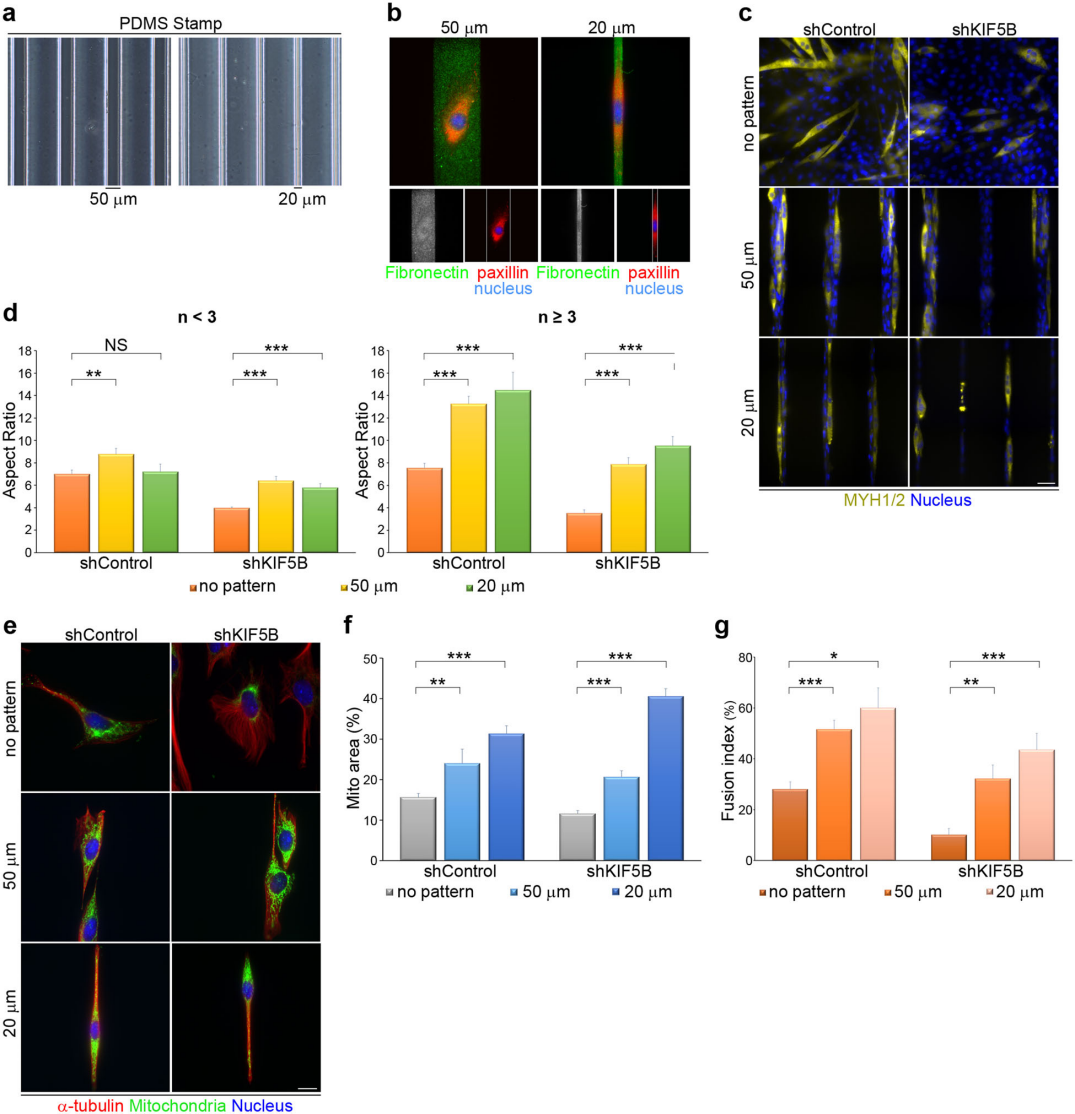
Geometric cue-coordinated cell adhesions are able to rescue the myogenic defects caused by kinesin-1 inhibition. **a** A polydimethylsiloxane (PDMS) stamp with micron-sized features (20 µm or 50 µm stripes) were shown in phase images. **b** Images of C2C12 cells plated on 20 µm or 50 µm stripe micropatterns; these were stained for fibronectin (to visualize stripe micropatterns; green), paxillin (to visualize FAs; red) and DAPI (to visualize nucleus; blue). **c** C2C12 cells expressing non-silenced (shControl) or kinesin-1-silenced (shKIF5B) shRNAs were plated on fibronectin-coated coverslips (with no pattern substrate), 20 µm striped coverslips or 50 µm striped coverslips, treated with differentiation medium for 5 days and immunostained for MYH1/2 (yellow; myotube marker) and DAPI (to visualize nucleus). Scale bar, 50 µm. **d** Aspect ratio, calculated as a ratio of the longest cell length to the shortest cell length in MYH1/2^+^ cells with nuclei <3 (*n* < 3) or nuclei ≥3 (*n* ≥ 3), as shown in **c**. Data are mean ± s.e.m (shControl with no pattern with <3 nuclei, *n* = 68 MYH1/2^+^ cells; shControl on 50 µm stripes with <3 nuclei, *n* = 34 MYH1/2^+^ cells; shControl on 20 µm stripes with <3 nuclei, *n* = 24 MYH1/2^+^ cells; shKIF5B with no pattern with <3 nuclei, *n* = 168 MYH1/2^+^ cells; shKIF5B on 50 µm stripes with <3 nuclei, *n* = 65 MYH1/2^+^ cells; shKIF5B on 20 µm stripes with <3 nuclei, *n* = 38 MYH1/2^+^ cells; shControl with no pattern with ≥3 nuclei, *n* = 45 MYH1/2^+^ cells; shControl on 50 µm stripes with ≥3 nuclei, *n* = 52 MYH1/2^+^ cells; shControl on 20 µm stripes with ≥3 nuclei, *n* = 21 MYH1/2^+^ cells; shKIF5B with no pattern with ≥3 nuclei, *n* = 23 MYH1/2^+^ cells; shKIF5B on 50 µm stripes with ≥3 nuclei, *n* = 25 MYH1/2^+^ cells; shKIF5B on 20 µm stripes with ≥3 nuclei, *n* = 23 MYH1/2^+^ cells). **p < 0.01; ***p < 0.001; NS no significance. **e** C2C12 cells expressing non-silenced (shControl) or kinesin-1-silenced (shKIF5B) shRNAs were plated on fibronectin-coated coverslips (with no pattern substrate), 20 µm striped coverslips or 50 µm striped coverslips for 24 h and immunostained for Tom20 (green; to visualize mitochondria), α-tubulin (red; to visualize microtubules), and DAPI (to visualize nucleus). Scale bar, 50 µm. **f** Percentage of mitochondria area (marked with Tom20) within a cell (marked with α-tubulin), as shown in **e**. Data are mean ± s.e.m (shControl with no pattern, *n* = 32 cells; shControl on 50 µm stripes, *n* = 22 cells; shControl on 20 µm stripes, *n* = 20 cells; shKIF5B with no pattern, *n* = 39 cells; shKIF5B on 50 µm stripes, *n* = 34 cells; shKIF5B on 20 µm stripes, *n* = 20 cells). **p < 0.01; ***p < 0.001. **g** Fusion index, calculated as the percentage of nuclei (≥3) in MYH1/2^+^ cells, as shown in **c**. Data are mean ± s.e.m (shControl with no pattern, *n* = 154 MYH1/2^+^ cells; shControl on 50 µm stripes, *n* = 112 MYH1/2^+^ cells; shControl on 20 µm stripes, *n* = 53 MYH1/2^+^ cells; shKIF5B with no pattern, *n* = 165 MYH1/2^+^ cells; shKIF5B on 50 µm stripes, *n* = 108 MYH1/2^+^ cells; shKIF5B on 20 µm stripes, *n* = 56 MYH1/2^+^ cells). *p < 0.05; **p < 0.01; ***p < 0.001.

The above results show that, in the presence of RBL, either exogenous auto-clustering integrin β1 or constitutively active integrin β1 is down-regulated and is not able to restore myogenic defects (Fig. 4f). We then hypothesized that kinesin-1 mediates integrin β1 transport to transmit the FA-related signals that not only shape cells into an elongated morphology, but also deliver the signals needed for myogenic differentiation. In order to examine further whether kinesin-1 disruption causes myogenic defects via a reduction in cell adhesion (which is integrin β1 - dependent), we used microcontact printing techniques (see Materials and methods section) to generate stripes of fibronectin on polydimethylsiloxane (PDMS) substrate (Fig. 5a, b); the width of these stripes was set to either 50 µm or 20 µm. Non-silenced and kinesin-1-silenced cells were then plated on fibronectin-coated coverslips (with no pattern substrate), 20 µm striped coverslips or 50 µm striped coverslips; these cells were then cultured in differentiation media. After 5 days, cells were stained with MYH1/2 protein and nuclei (Fig. 5c). We then quantified the aspect ratio of the identified MYH1/2^+^ cells, and found that both 20 µm and 50 µm fibronectin-coated stripes were able to shape the kinesin-1-silenced cells into an elongated morphology, including both cells with fewer than three nuclei and cells with three or more nuclei (Fig. 5d). An assessment of the distribution of mitochondria in the above three types of cells, using Tom 20 staining (Fig. 5e), revealed that the reduction in spreading area of the mitochondria caused by kinesin-1 depletion was rescued when the kinesin-1-silenced cells were plated on either 20 µm or 50 µm fibronectin-coated stripes (Fig. 5f). Next, we measured the myogenic fusion indices of the various types of cells and the results confirmed that the 20 µm and 50 µm fibronectin-coated stripes significantly restored the defect in multi-nuclei myotube formation found in kinesin-1-silenced cells (Fig. 5g). Therefore, it is clear that the fibronectin-coated stripes were able to elongate the cells, and that cell adhesion-mediated cell polarity can restore the defects (caused by kinesin-1 depletion), including mitochondrial distribution and myogenic differentiation.

## Discussion

**Fig. 6:**
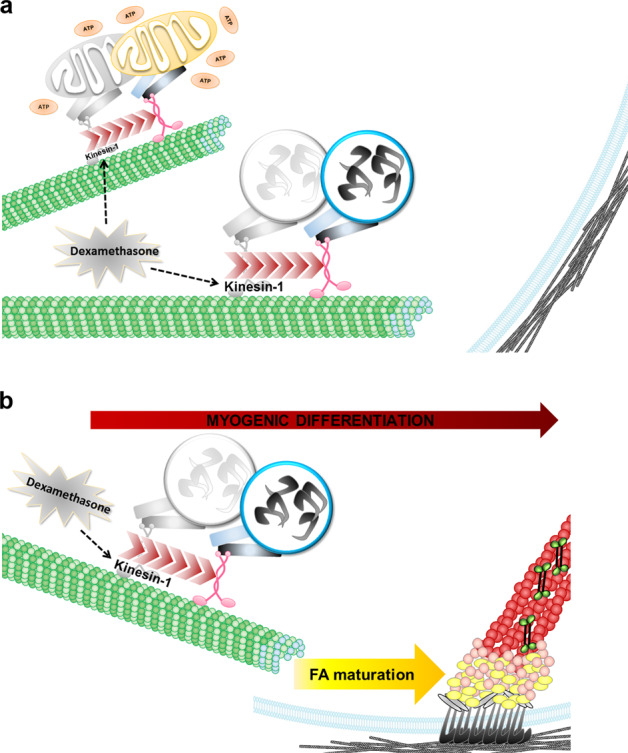
Model of the effects of dexamethasone-enhanced kinesin-1 activity on focal adhesion maturation and thence myogenic differentiation. **a** Upon dexamethasone treatment, the microtubule motor kinesin-1 moves faster, which facilitates mitochondria movement and integrin β1 translocation to the plasma membrane. **b** Kinesin-1-facilitated integrin β1 translocation, bringing about focal adhesion maturation, which in turn contributes to myogenic differentiation.

The goal of this study was to clarify the mechanism by which Dex regulates myogenic differentiation. To achieve this goal, we first used an in vitro kinesin-1 activity assay to confirm the direct effect of Dex on kinesin-1 motors activation. Upon Dex stimulation, the speed of kinesin-1 moving along the microtubules was accelerated, which also increased mitochondrial dispersion (Fig. 6a). In mice with muscle injury, we observed that Dex treatment promoted the regeneration of skeletal muscles via kinesin-1 motor activity (Fig. 2). Therefore, Dex is likely to regulate muscle differentiation by activating kinesin-1. These observations highlight the importance of exploring how kinesin-1 activated by Dex affects myogenic differentiation. Indeed, we confirmed that, during myogenic differentiation, kinesin-1 was responsible for the transport of integrin β1 along the microtubules to the plasma membrane, which in turn increased clustering of integrin β1 on plasma membrane. The activation and clustering of integrin β1 resulted in FA assembly, FA maturation, and the transmission of integrin β1-mediated FA signals that in turn facilitated myogenic differentiation (Fig. 6b). These findings indicate, for the first time, that Dex is able to directly activate kinesin-1 motor proteins, thereby enhancing myogenic differentiation via FA signaling mediated by integrin β1. Clarifying the important role of kinesin-1 in Dex-mediated myogenic differentiation has the potential to become an important therapeutic strategy when clinically managing muscle regeneration.

Previous studies have demonstrated that various glucocorticoids, including Dex, are able to affect mitochondrial energy production via both mitochondrial respiration and gluconeogenesis^55^. These metabolic states are modified by mitochondrial morphological adaptation, especially by dynamic events such as transport, fusion, fission, and quality control. The morphological change of mitochondria serves as a regulator in the process of myogenic differentiation^44^. In our study, we examined the positive effect of Dex and its beneficial role in myogenic differentiation (Supplemental Fig. 1a). We also found that mitochondrial transport was accelerated by Dex, and, in particular, that its transport speed was microtubule-dependent (Fig. 1c, d). Mitochondria are linked to the microtubule motor protein kinesin-1^48^ via the adapter protein Milton and the mitochondrial membrane GTPase miro^56,57^, and thereby providing the possibility that Dex is able to act directly on the kinesin-1 motors. The assertion of this hypothesis was confirmed by in vitro kinesin-1 motility assays. By measuring the movement speed of rhodamine-labeled microtubules, we confirmed that Dex directly promoted kinesin-1 motor activity and accelerates the kinesin-1 movement along the microtubules (Fig. 1e, f). Next, we used kinesin-1-silenced C2C12 cells, and found that Dex treatment resulted in blocking of the increased mitochondrial movement (Fig. 3b), the mitochondrial dispersion (Fig. 3c, d), and the myogenic differentiation (Fig. 3e–g). These findings support the notion that kinesin-1 is required for mitochondrial transport^48^ and myogenic differentiation^32^. Altogether, we conclude that Dex is able to promote myogenic differentiation via a direct enhancement of kinesin-1 motility.

Integrin β1-mediated FA signaling regulates myogenic differentiation^29,30^. Several studies have revealed that the major cell-ECM adhesive molecules in muscle are integrin β1 with its different α subunits^58^; these are known to be required for myoblast fusion and sarcomere assembly^30^. Mice with an integrin β1 deficiency in skeletal muscle die at birth with non-inflated lungs because of their inherent muscle defects, which supports the importance of integrin β1 in muscle development^30^. Integrin β1-deficient muscle fibers lack a typical striate pattern and also show defects in the lateral linkage of the muscle fiber cytoskeleton to the sarcolemma^30^; these defects are similar to those found in kinesin-1-deficient skeletal muscle^48^. Despite the fact that the effects of integrin β1 and kinesin-1 in muscle development have been investigated previously, our study has revealed, for the first time, a novel mechanism whereby kinesin-1 is able to regulate integrin β1 activation at FAs (Fig. 4b, c) and for FA signals transmission (Fig. 4d, e). We have confirmed that integrin β1 is transported along microtubules via kinesin-1 motors during myogenic differentiation (Fig. 4g), which explains why auto-clustering integrin β1 (integrinβ1 V737N) and constitutively active integrin β1 (integrinβ1 G429N) are unable to restore the myogenic defects caused by RBL treatment (Fig. 4f). By plating C2C12 cells onto fibronectin-coated microstripes, and thereby forcing integrin β1 activation in cells with polarized morphology, the defects caused by kinesin-1 disruption in mitochondria distribution (Fig. 5e, f) and myogenic differentiation (Fig. 5g) can be rescued. This new finding indicates that integrin β1 activation plays a crucial role in driving the myogenic machinery, and that this is regulated by microtubule motors kinesin-1.

The binding of kinesin-1 motor proteins to the microtubules is crucial to kinesin-1-driven transport, which is able to haul various molecular cargoes to specific reaction sites in cells. The binding influences microtubule stability. When kinesin-1 shows strong binding to microtubules, it inhibits the shrinkage of microtubules and enhances the stability of their microtubule tracks. In the absence of kinesin binding, microtubules rapidly depolymerized^59^. We have shown that treatment with RBL inhibits kinesin-1 activity by blocking the binding between kinesin-1 and microtubules^50^, which, in turn, suppresses the protein levels of kinesin-1 and α-tubulin in C2C12 cells (Fig. 3i). A previous study has revealed an enhancement of ubiquitinated c-MYC, p53, kinesin-1, and α-tubulin in RBL-treated HEK293T cells^51^; thus, we assume that, in RBL-treated C2C12 cells, kinesin-1 and α-tubulin are degraded via the proteasome pathway. Interestingly, we have also shown that the protein level of GFP-integrin β1 wt, GFP-integrin β1 V737N (the auto-clustering integrin β1), and GFP-integrin β1 G429N (constitutively active integrin β1) decreases in the cells treated with RBL (Fig. 4f). Since we have confirmed the effect of kinesin-1 on integrin β1 transport along microtubules for FAs assembly and FA signals transmission, the dissociation of kinesin-1 from microtubules by RBL may cause integrin β1 to deviate from its normal path and undergo proteasomal degradation. Therefore, further studies are needed to determine how kinesin-1 mediates integrin β1 transport and how kinesin-1 activity controls the proteasomal degradation of integrin β1.

## Materials and methods

### Cell culture

The mouse myoblasts C2C12 were kindly provided by Dr. Pei-Ching Chang (National Yang Ming Chiao Tung University, Taiwan). Cells have been tested and confirmed to be free of mycoplasma contamination. Cells were cultured in DMEM-high glucose (ThermoFisher) supplemented with 10% FBS (ThermoFisher) and 1% penicillin/streptomycin (ThermoFisher). C2C12 differentiation was carried out using myogenesis induction medium (DMEM-high glucose supplemented with 2% horse serum and 1% penicillin/streptomycin). C2C12 cells stably expressing non-silenced and KIF5B shRNA were generated using a lentiviral shRNA system according to the manufacturer’s instructions (National RNAi Core Facility Platform/Academia Sinica). Transient transfections were performed using Lipofectamine 2000 (ThermoFisher). For all experiments, the cells were seeded on fibronectin-coated coverslips, plates, or microstripes.

### Plasmids and reagents

#### Plasmids

Expression silencing of kinesin-1 proteins was achieved using pLKO-KIF5B shRNA (TRCN0000332767, National RNAi Core Facility Platform). pLKO vector (TRCN000208001, National RNAi Core Facility Platform) was used as a control (non-silenced shRNA). The GFP-integrin β1 wt, GFP-integrin β1 V737N, and GFP-integrin β1 G429N were kindly provided by Dr. Ming-Jer Tang (National Cheng Kung University, Taiwan).

#### Antibodies

Mouse anti-paxillin (BD 610052); rat anti-active integrin β1 (9EG7; BD 553715); rabbit-anti-Tom20 (Santa Cruz sc-11415); mouse-anti-MYH1/2 (Santa Cruz sc-53088); rabbit anti-fibronectin (Santa Cruz sc-9068); rabbit anti-GAPDH (GeneTex GTX100118); rabbit anti-β-actin (GeneTex GTX100313); rabbit anti-integrin β1 (GeneTex GTX128839); rabbit anti-paxillin (GeneTex GTX125891); rabbit anti-pY397-FAK (GeneTex GTX129840); rabbit anti-kinesin-1 (Abcam AB167429); rabbit anti-GFP (Abcam AB290); mouse anti-α-tubulin (Sigma T5168); mouse anti-MYH2 (Thermo Fisher 14-6503-80); rabbit anti-FAK (Thermo Fisher AHO0502); Alexa Fluor 488 phalloidin (Thermo Fisher A12379); Alexa Fluor 568 phalloidin (Thermo Fisher A12380); Alexa Fluor 488-anti-rabbit IgG (Thermo Fisher A11034); Alexa Fluor 488-anti-mouse IgG (Thermo Fisher A11029); Alexa Fluor 488-anti-rat IgG (Thermo Fisher A11006); Alexa Fluor 568-anti-rabbit IgG (Thermo Fisher A11036); Alexa Fluor 568-anti-mouse IgG (Thermo Fisher A11031); DAPI (Thermo Fisher D1306); Mitotracker Red (Thermo Fisher M7512); HRP-AffiniPure mouse anti-rabbit IgG (Jackson ImmunoResearch 211-032-171); and HRP-AffiniPure goat anti-mouse IgG (Jackson ImmunoResearch 115-035-174).

#### Reagents

Nocodazole (Sigma); Dexamethasone (Dex; Sigma); Rose Bengal Lactone (RBL; Sigma); and Barium Chloride (BaCl_2_; Sigma).

### RNA extraction, reverse transcription, and real-time quantitative PCR

RNA was extracted from cells using TRIzol reagent (Invitrogen) and total RNA precipitated according to the manufacturer’s instructions. The RNA products were reverse-transcribed using RevertAid First Strand cDNA Synthesis Kits (ThermoFisher) and random hexamer primers. The cDNA products were amplified by PCR using a KAPA SYBR® FAST qPCR Kits (ABI Prism; Roche). The 18SrRNA gene and cytochrome C oxidase I gene were used as indicators of nuclear DNA and mitochondrial DNA, respectively. Quantification of the target mRNA was carried out by the ΔΔCT method. The qPCR primers for the nuclear 18S rRNA gene were 5′-TAGAGGGACAAGTGGCGTTC-3′ and 5′-CGCTGAGCCAGTCAGTGT-3′. The qPCR primers for the mitochondrial cytochrome C oxidase I gene were 5′-GCCCCAGATATAGCATTCCC-3′ and 5′-GTTCATCCTGTTCCTGCTCC-3′.

### Measurement of ATP production rate

ATP production rate was calculated from oxygen consumption rate (OCR) using a Seahorse XF^e^24 Extracellular Flux Analyzer (Seahorse Bioscience) according to the manufacturer’s protocol. C2C12 cells were seeded in culture medium 24 h before measurement at a density of 3 × 10^4^ cells per well, and then treated with DMSO or 1 μM Dex for 6 h for analysis. Oligomycin (1 µM), Carbonyl cyanide 4-(trifluoromethoxy)phenylhydrazone (FCCP; 1 µM), and antimycin A (0.5 µM) with rotenone (0.5 µM) were added sequentially to evaluate mitochondrial respiration. The data were normalized by the amount of protein present in each well.

### Kinesin-1 motility assay

The protocol for kinesin-1 motility assay was followed according to manufacturer’s instructions (BK027; Cytoskeleton). To prepare tubulin stock proteins, 60 μg rhodamine tubulin (TL331M; Cytoskeleton) in 12 μl ice-cold General Tubulin Buffer (BST01-001; Cytoskeleton) were mixed with 250 μg unlabeled tubulin (TL238-A; Cytoskeleton) in 50 μl ice-cold General Tubulin Buffer, and further mixed with 12 μl ice-cold Microtubule Cushion Buffer (BK027-CB; Cytoskeleton). Immediately, the mixed proteins were split into 2 μl aliquot, placed in individual eppendorf tube and frozen in liquid nitrogen for storage at −70 °C. For the preparation of polymerized microtubule, 2 μl of tubulin stock proteins were mixed with 2 μl ice-cold Microtubule Cushion Buffer for 15 min at 35 °C. Subsequently, 100 μl Taxol supplemented General Tubulin Buffer (20 μM Taxol in General Tubulin Buffer) were added, and gently mixed. This step results in a population of Taxol-stabilized microtubules with an average length of 5 to 10 μm and at a concentration of 7 × 10^10^/ml. To remove the un-polymerized tubulin to decrease the background fluorescence, 104 μl of microtubule solution were carefully layered onto 400 μl Taxol supplemented Microtubule Cushion Buffer (20 μM Taxol in Microtubule Cushion Buffer) into an ultracentrifugation tube (Beckman), and centrifuged at 100,000×*g* at 25 °C for 30 min to pellet the microtubules. The microtubule pellets were gently re-suspended in 100 μl Taxol supplemented General Tubulin Buffer. The Taxol-stabilized microtubule stock population remains stable at room temperature for several hours. To perform the kinesin-1 motility assay, the kinesin-1 stock proteins were prepared by mixing 25 µg recombinant kinesin-1 motor domain proteins (KR01; Cytoskeleton) in 6 µl Kinesin Buffer (BK027-KB; cytoskeleton), which was then split into 1 μl aliquot in individual eppendorf tubes and then frozen in liquid nitrogen for storage at −70 °C. One tube of kinesin-1 stock proteins was mixed with 11 µl General Tubulin Buffer, which were perfused in the acid washed perfusion chamber (BSM05-04; Cytoskeleton), and incubated at room temperature for 5 min. This was followed by blocking of the perfusion chamber with 11 µl blocking solution (BK027-BL; Cytoskeleton) at room temperature for 5 min; 10 μl fluorescent Taxol-stabilized microtubule stock was then perfused into the perfusion chamber and incubate for 5 min at room temperature. Subsequently, 20 μl Wash buffer solution [17.8 μl Chamber Wash Buffer (BK027-WB; Cytoskeleton), 0.2 µl 2 mM taxol and 2 µl 10x antifade] was perfused into the chamber to remove any microtubules that were not bound to the kinesin-1 motor proteins. To activate the motility of the kinesin-1 motor proteins, 10 µl motility buffer (4.2 µl General Tubulin Buffer, 4.2 µl Chamber Wash Buffer, 0.1 µl 2 mM taxol, 1 µl 10X antifade, and 0.5 µl 100 mM ATP) supplemented with DMSO or 1 µM Dex was perfused into the chamber. The time-lapse epi-fluorescence images were obtained using a microscope (DMRBE, Leica) coupled with a 100XNA1.3 PL FLUOTAR objective lens (Leica) and an 512B EMCCD (Andor); these were operated by Micro-Manager 1.4 software (Leica). The time-lapse images showing microtubule movement were analyzed using Metamorph in order to assess the kinesin-1 motor activity.

### Microtubule isolation

C2C12 cells were plated on culture dishes coated with 10 µg/ml fibronectin for 24 h at 50% confluence. The cells were then washed twice with PBS at 37 °C and incubated with microtubule-stabilizing buffer (100 mM PIPES, pH 6.9, 5 mM MgCl_2_, 2 mM EGTA, 2 M glycerol, 0.1% NP40, 10 mM beta-glycerophosphate, 50 mM NaF, 0.3 μM okadaic acid, and 1 mM PMSF) containing protease inhibitors and phosphatase inhibitors (Roche) for 15 min at 37 °C. Cell lysate was collected by scraping with a rubber policeman, and the suspension centrifuged at room temperature for 5 min at 1000 × *g*. After centrifugation, the supernatants (soluble fraction) were collected, and the pellets (insoluble fraction) were solubilized and sonicated in microtubule-stabilizing buffer for 15 s on ice.

### Time-lapse tracking to assess mitochondrial dynamics and related image analysis

To analyze the dynamics of mitochondria, cells were stained with 25 nM Mitotracker Red and mounted on a magnetic chamber (LCI) cultured in phenol red-free culture medium with 25 mM Hepes (pH = 7.4). Time-lapse confocal images of Mitotracker Red were captured at 2 s intervals using an *iLas* multi-modal of TIRF (Roper)/spinning disk confocal (CSUX1, Yokogawa) microscope (Ti-E, Nikon) system equipped with 100 × 1.49NA Plan objective lens (Nikon) and an EMCCD (ProEM, Princeton). To track and analyze the dynamics of Mitotracker Red, a mitochondria-tracking program Mytoe^47^ was used to quantified mitochondria movement (mitochondria speed). The results are presented graphically using Excel software (Microsoft).

### Immunofluorescence analysis and image analysis

For paxillin/active integrin β1 (9EG7) staining, the cells were fixed with 4% paraformaldehyde in cytoskeleton buffer (10 mM MES pH 6.1, 138 mM KCl, 3 mM MgCl_2_, and 2 mM EGTA) at room temperature for 20 min, permeabilized with cytoskeleton buffer containing 0.5% Triton X-100 at room temperature for 5 min, removed aldehyde groups with 0.1 M glycine in PBS at room temperature for 10 min, and blocked with blocking solution (3% BSA/0.02% Triton-X100 in PBS) at room temperature for 60 min. Subsequently, the cells were incubated with the indicated primary antibodies in blocking solution at 4 °C for 16 h, and then incubated with fluorescent dye–conjugated secondary antibody at room temperature for 1 h. For mitochondria (Tom20) and MYH staining, the cells were fixed with 4% paraformaldehyde at room temperature for 20 min, permeabilized with PBS containing 0.1% saponin at room temperature for 5 min, removed aldehyde groups with 0.1 M glycine in PBS at room temperature for 10 min, and blocked with blocking solution (2% BSA in PBS) at room temperature for 1 h. Subsequently, the cells were incubated with the indicated primary antibodies in blocking solution at 4 °C for 16 h, and then incubated with fluorescent dye–conjugated secondary antibody at room temperature for 1 h. Last, the cells were mounted on coverslips using fluorescent mounting medium (DAKO), or on a magnetic chamber (LCI) and incubated with PBS containing *N*-propyl gallate for epi-fluorescence or TIRF imaging, respectively.

TIRF images were obtained using an *iLas* multi-modal of TIRF (Roper)/spinning disk confocal (CSUX1, Yokogawa) microscope (Ti-E, Nikon) system equipped with 60 × 1.49NA or 100 × 1.49NA Plan objective lens (Nikon) on an Evolve EMCCD (Photometrics) with an ~100 nm evanescent field depth. TIRF images were captured and processed using Metamorph software. Epi-fluorescence images were obtained using a microscope (DMRBE, Leica) coupled with a 40 × NA1.0 or 63 × NA1.4 objective lens (Leica) and an 512B EMCCD (Andor); these were operated by Micro-Manager 1.4 software (Leica). Slides-scanned epi-fluorescence images were obtained using an epi-fluorescence microscope system (Ti-E, Nikon) coupled with a 40XNA1.3 or 100XNA1.49 Plan objective lens (Nikon) and a sCMOS camera (OHCA-Flash 4.0, 1024 × 1024 pixels, Hamamatsu); these were operated by NIS-Elements software (Nikon). H&E staining of the samples of tibialis anterior (TA) muscles were obtained using an epi-fluorescence microscope system (TS100-F, Nikon) coupled with a 40XNA0.6 objective lens (Nikon) and a Whited WS-500 CCD camera.

### Fabrication of the micropatterned substrates

The protocol for the fabrication of micropatterned substrates on PDMS was carried out as described previously^60^. Briefly, PDMS stamps (linear fabricated stamps) were cast, baked, and removed from their silicon wafers (master templates), which was fabricated by photolithographic methods. The surface of the PDMS stamps were coated with fibronectin (50 µg/ml) for 1 h, washed with sterilized ddH_2_O, and dried with compressed air. A glass coverslip spin-coated with PDMS was UV oxidized for 10 min (UVO cleaner 42; Jelight), and then placed in contact with a PDMS stamps for 2 min in order to transfer the fibronectin. The coverslips were then blocked with 0.2% Pluronic® F-127 (Sigma) for 1 h, and rinsed three times with PBS before cell seeding.

### Analysis of muscle regeneration

For regeneration studies, tibialis anterior muscles in the left hind legs of 9-week-old C57BL/6 females mice were injured by intramuscular injection of 60 µl 1.2% BaCl_2_. All mice were randomly divided into three groups with five mice per group. Following the intraperitoneal injection of DMSO, Dex (0.1 mg/kg) or Dex (0.1 mg/kg) + RBL (30 mg/kg) were administered everyday. The muscles were collected at the indicated time points, and embedded in paraffin for microtom sectioning. Regeneration after injury was analyzed in the hematoxylin and eosin (H&E)-stained muscle sections by measuring the number of centrally nucleated regenerating myofibers within the injured area. The centrally nucleated regenerating myofibers in 20X magnification field. For each sample, five fields were randomly selected and counted. The animal care and experimental protocols were approved by the Institutional Animal Care and Use Committee (IACUC) of National Yang Ming Chiao Tung University. The mice were housed on a 12-h light and 12-h dark cycle.

### Statistical analysis and data presentation

Statistical significance was calculated by either the Student’s *t* test or one-way ANOVA. All the graphs were plotted using Excel software (Microsoft).

## Supplementary information

Supplemental Figure 1

Supplemental Figure 2

Supplemental Figure Legends
