# Supplementary figures and images for: Dexamethasone accelerates muscle regeneration by modulating kinesin-1-mediated focal adhesion signals
Source: Cell Death Discov. 2021 Feb 17;7:35. doi: 10.1038/s41420-021-00412-4 (PMC7889929; doi:10.1038/s41420-021-00412-4)

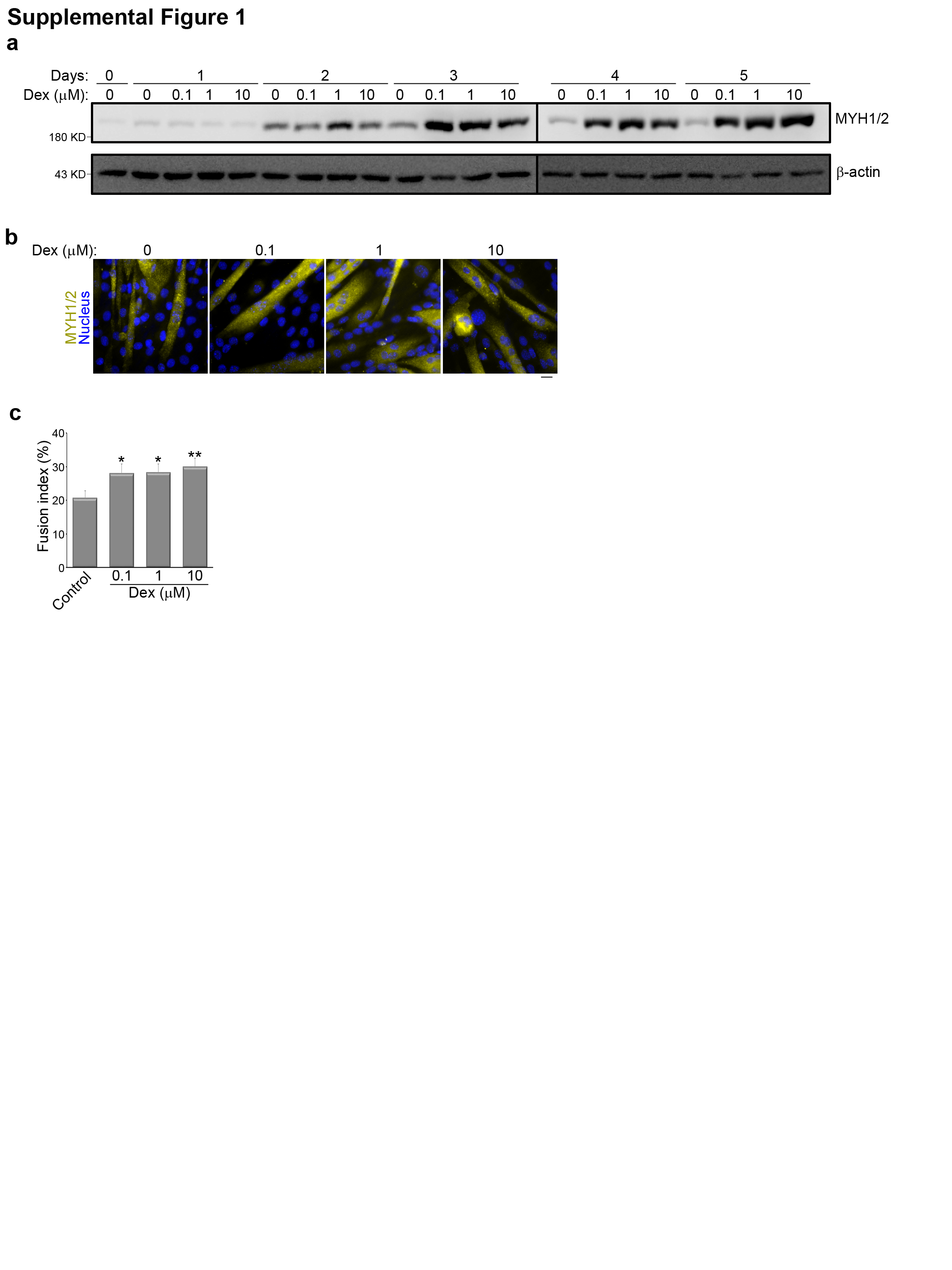

Supplement: Supplementary file 1 — Supplemental Figure 1 [file 41420_2021_412_MOESM1_ESM.tif]

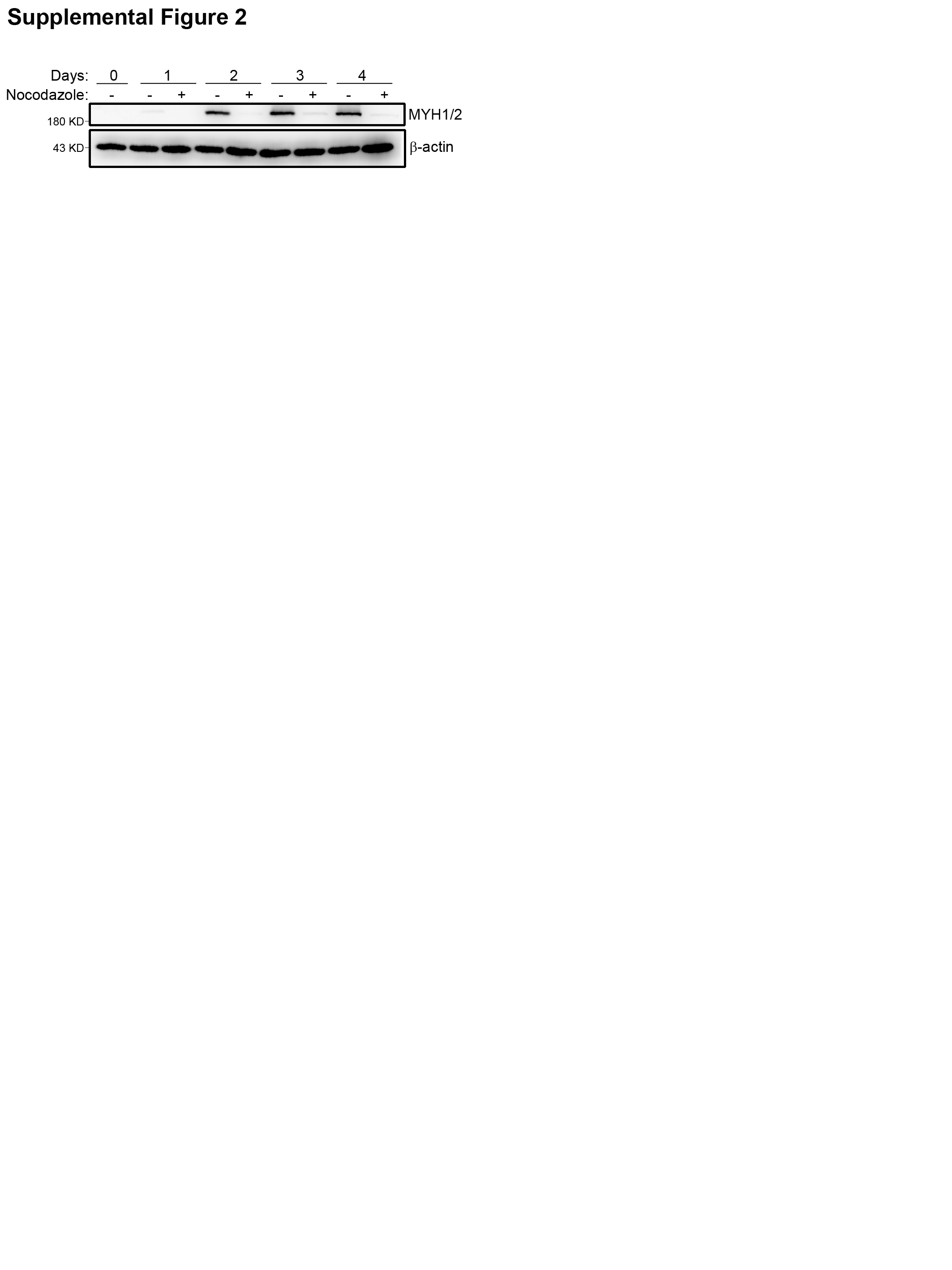

Supplement: Supplementary file 2 — Supplemental Figure 2 [file 41420_2021_412_MOESM2_ESM.tif]
