# Supplementary material for: Dexamethasone accelerates muscle regeneration by modulating kinesin-1-mediated focal adhesion signals
Source: Cell Death Discov. 2021 Feb 17;7:35. doi: 10.1038/s41420-021-00412-4 (PMC7889929; doi:10.1038/s41420-021-00412-4)
Supplement: Supplementary file 3 — Supplemental Figure Legends [file 41420_2021_412_MOESM3_ESM.doc]

**Lin_Supplemental Figure Legends**

**Figure S1. Dexamethasone enhances myogenic differentiation.**

(a) Effects of Dex on myogenic differentiation. C2C12 cells were cultured in a myogenic differentiation medium with 0, 0.1, 1 and 10 µM Dex for 0, 1, 2, 3, 4 and 5 days. The protein level of MYH1/2 and β-actin in the cell lysate (loaded with equal amounts of total protein) were detected by Western blotting. (b) Images of immunostained MYH1/2 (yellow; myogenic marker) and DAPI (to visualize nucleus) in C2C12 cells cultured in a myogenic differentiation medium with 0, 0.1, 1 and 10 µM Dex for 5 days. Scale bar, 20 μm. (c) Fusion index, calculated as the percentage of nuclei (≥ 3) in MYH1/2+ cells from the images in (b). Data are mean±s.e.m (0µM Dex, n = 14 MYH1/2+ cells; 0.1µM Dex, n = 14 MYH1/2+ cells; 1µM Dex, n = 14 MYH1/2+ cells; 10µM Dex, n = 12 MYH1/2+ cells). **p*<0.05; ***p*<0.01.

**Figure S2. The Growth of microtubule correlates with myogenic differentiation.**

How the growth of microtubules affects myogenic differentiation. C2C12 cells were cultured in a myogenic differentiation medium with 10 µM Nocodazole for 0, 1, 2, 3 and 4 days. The protein level of MYH1/2 and β-actin in the cell lysate (loaded with equal amounts of total protein) were detected by Western blotting.
